# The Efficacy of N-acetylcysteine as an Adjunct in the Management of Acute Zinc Phosphide Poisoning: A Randomized Double-Blind Controlled Trial

**DOI:** 10.7759/cureus.110440

**Published:** 2026-06-08

**Authors:** Siva K V, Jahnu Bhoj Nagal, Kuldeep Meena, Kavita Jain

**Affiliations:** 1 Department of Emergency Medicine, Jawaharlal Nehru Medical College and Hospital, Ajmer, IND

**Keywords:** acute zinc phosphide poisoning, in-hospital mortality, n-acetylcysteine, randomized controlled trial, vasoactive support

## Abstract

Background and objective

Acute zinc phosphide poisoning is a life-threatening toxicological emergency with high mortality, primarily caused by phosphine-induced oxidative stress and mitochondrial dysfunction. In the absence of a specific antidote, identifying effective adjunctive therapies is critical. This study aimed to evaluate whether intravenous N-acetylcysteine (NAC) can improve clinical outcomes when added to standard supportive care.

Methods

A prospective, randomized, double-blind, placebo-controlled trial was conducted in a single tertiary care emergency department in Northwest India from April 2024 to March 2025. This trial was registered with the Clinical Trials Registry - India (CTRI/2024/03/064278). Sixty adults (18-70 years) with confirmed zinc phosphide poisoning were randomly assigned to receive either standard supportive care with intravenous NAC (300 mg/kg over 20 hours) or standard care with a 5% dextrose placebo. Baseline demographic and clinical characteristics were comparable between the two groups. Severity at presentation, assessed by the requirement for inotropic support at admission, was also comparable between groups, with nine patients (30.0%) in the NAC group and eight patients (26.7%) in the placebo group requiring inotropic support at baseline (p = 0.83). The primary outcome was in-hospital mortality. Secondary outcomes included need for vasoactive support at admission and 24 hours, and changes in systolic blood pressure, arterial potential of hydrogen (arterial pH), alanine aminotransferase (ALT), aspartate aminotransferase (AST), and creatine kinase-MB isoenzyme (CK-MB).

Results

Baseline characteristics were comparable between groups. The NAC group showed lower in-hospital mortality than the placebo group (6.7% vs. 20.0%; Fisher’s exact p = 0.254; relative risk (RR): 0.33, 95% confidence interval (CI): 0.07-1.52; absolute risk reduction (ARR): 13.3%) and reduced requirement for inotropic support at 24 hours (6.7% vs. 23.3%; Fisher’s exact p = 0.145; RR: 0.29, 95% CI: 0.07-1.27; ARR: 16.6%); however, neither difference reached statistical significance. Serum ALT and AST levels were significantly lower in the NAC group than in the placebo group, with median ALT values of 92 IU/L versus 205 IU/L and median AST values of 48 IU/L versus 150 IU/L, respectively (p < 0.001 for both). No significant differences were observed in arterial pH or CK-MB levels. No adverse effects related to NAC were reported.

Conclusions

In this preliminary single-center trial, adjunctive intravenous NAC was not associated with a statistically significant reduction in in-hospital mortality or the requirement for inotropic support; however, a clinically meaningful trend was observed. Larger multicenter randomized trials are needed to determine the role of NAC in acute zinc phosphide poisoning.

## Introduction

Zinc phosphide is a common rodenticide and agricultural fumigant, particularly in rural areas of Southeast Asia and the Indian subcontinent. It is inexpensive, readily accessible, and highly toxic, making it a frequent agent in self-harm and a major public health concern in developing countries [1,2]. Aluminum phosphide poisoning has also been widely reported in agricultural regions and shares a similar phosphine-mediated toxicity profile [3,4]. Phosphide poisoning accounts for a substantial proportion of toxicological emergencies presenting to emergency departments in India, with mortality rates ranging from 20 to 50% in severe cases [5]. Once ingested, zinc phosphide reacts with gastric acid and moisture to release phosphine gas, a potent toxin that is rapidly absorbed into the bloodstream.

Phosphine exerts its major toxic effects by inhibiting cytochrome c oxidase, a key component of the mitochondrial electron transport chain, thereby disrupting oxidative phosphorylation and shifting cellular metabolism toward an anaerobic pathway [6]. Phosphide poisoning is a rapidly progressive toxicological emergency, with severe cardiovascular collapse, metabolic acidosis, hepatic injury, and multiorgan dysfunction reported in critical cases [7,8]. The excessive production of reactive oxygen species causes lipid peroxidation and extensive oxidation of cellular proteins and DNA [9]. Oxidative stress consistently triggers cellular energy loss, triggers inflammatory mechanisms, and eventually leads to the malfunctioning of various organs [10].

The clinical presentation is characterized by predominant gastrointestinal symptoms, including nausea, vomiting, and abdominal pain, which can rapidly progress to moderate or severe poisoning. Systemic complications include acute liver injury, severe metabolic acidosis, renal dysfunction, and profound cardiovascular instability [5]. Refractory shock, often associated with complex dysrhythmias and myocardial depression, is the most common cause of death [7]. Laboratory abnormalities such as elevated transaminase levels, metabolic acidosis, and coagulopathy provide important indicators of toxicity severity and are associated with a poor prognosis [6].

There is no specific antidote for zinc phosphide poisoning, and treatment relies largely on aggressive supportive care. Current management strategies are centered on early resuscitation, correction of acid-base disturbances, vasopressor therapy, and organ-supportive measures [11,12]. The primary therapeutic goals are hemodynamic stabilization, correction of metabolic derangements, and the use of advanced organ-support interventions when required [1]. Because mitochondrial dysfunction and oxidative stress play central roles in the pathophysiology of phosphine toxicity, therapies that target these mechanisms have become an increasing focus of research [9].

N-acetylcysteine (NAC) is a well-established antioxidant with cytoprotective properties. It serves as a precursor to a key intracellular antioxidant, glutathione, and also acts as a direct scavenger of free radicals. Additionally, NAC modulates inflammatory signaling pathways and stabilizes mitochondrial membranes [10]. Studies have shown that NAC can prevent oxidative injury, preserve mitochondrial integrity, and inhibit apoptotic cell death in models of zinc phosphide toxicity [7]. These mechanistic effects provide a strong biological rationale for its use as an adjunctive therapy in such poisonings.

Zinc and aluminum phosphide poisoning share a similar phosphine-mediated mechanism of toxicity, although the present study specifically focuses on acute zinc phosphide poisoning. Clinical evidence supporting the use of NAC in phosphide poisoning is primarily derived from studies on aluminum phosphide, a structurally related compound that induces toxicity through a similar phosphine-mediated pathway [8]. Clinical trials and observational studies consistently show that adjunctive NAC therapy in aluminum phosphide poisoning is associated with improved survival, reduced vasopressor requirements, and decreased hepatic injury [13,14]. Its efficacy in mitigating cardiovascular toxicity and improving clinical outcomes in phosphide poisoning has also been reported in other studies [15,16]. Early reports suggest that similar benefits may extend to zinc phosphide exposure, particularly with prompt NAC administration [17,18].

Despite these promising findings, direct clinical evidence supporting the effectiveness of NAC in zinc phosphide poisoning remains limited. Although the biological rationale is compelling, the extrapolation of findings from aluminum phosphide studies requires validation through well-designed clinical trials. Given the high mortality associated with zinc phosphide poisoning and the absence of a specific antidote, there is a clear need to identify effective adjunctive therapeutic interventions. Therefore, this randomized, double-blind, placebo-controlled study was conducted to determine whether intravenous NAC, when added to standard supportive care, could improve clinical outcomes in patients with acute zinc phosphide poisoning.

This study aimed to evaluate the efficacy of intravenous NAC as an adjunct to standard supportive therapy in patients with acute zinc phosphide poisoning. The primary objective was to assess its effect on in-hospital mortality. Secondary objectives were to evaluate its impact on inotropic support requirements and selected physiological and biochemical parameters, including systolic blood pressure, arterial pH, alanine aminotransferase (ALT), aspartate aminotransferase (AST), and creatine kinase-MB (CK-MB).

## Materials and methods

Study design and setting

This was a prospective, randomized, placebo-controlled, double-blind interventional trial conducted in the emergency department of a single tertiary care academic hospital in Northwest India from April 2024 to March 2025. Ethical approval was obtained from the institutional ethics committee before study initiation (approval no.: 13954/Acad-III/MCA/20), and the study was conducted in accordance with standard ethical principles for biomedical research. Written informed consent was obtained from all participants or their legally authorized representatives. The trial was registered with the Clinical Trials Registry - India (CTRI/2024/03/064278). Although no formal interim analysis was planned, ongoing safety monitoring was maintained throughout the study period.

Study population and eligibility criteria

Adult patients aged 18-70 years with confirmed acute zinc phosphide poisoning were eligible for enrollment. Diagnosis was based on a clear history of zinc phosphide ingestion and a positive silver nitrate paper test of gastric aspirate. Patients were excluded if they were outside the specified age range, had co-ingestion of multiple or unidentified toxic agents, had pre-existing chronic systemic illnesses, including cardiac, hepatic, renal, or respiratory disease, or declined consent. No formal poisoning severity grading scale was applied, and therefore, severity-based subgroup analysis was not performed. Clinical severity at presentation was assessed using baseline hemodynamic status and evidence of organ dysfunction.

Randomization and blinding

Eligible participants were randomly assigned in a 1:1 ratio to either the NAC or placebo group using a computer-generated permuted block randomization sequence. Allocation concealment was ensured using sequentially numbered, sealed, opaque envelopes. To maintain double blinding, an independent pharmacist, who was not involved in patient care, data collection, or outcome assessment, prepared the study infusions. NAC and placebo infusions were supplied in identical, unlabeled 500 mL opaque intravenous bags with the same appearance, volume, and infusion schedule. Physicians, nursing staff, patients, and outcome assessors remained blinded to group allocation throughout the study. Group allocation was revealed only after database locking and completion of the primary analysis.

Intervention protocol

Patients in the intervention group received intravenous NAC at a total dose of 300 mg/kg over 20 hours. This regimen was chosen because it follows the standard intravenous NAC protocol commonly used in toxicology, has an established safety profile, and has been applied in comparable studies of phosphide poisoning [13,14,18]. The dosing schedule consisted of a loading dose of 150 mg/kg over 30-60 minutes, followed by 50 mg/kg over four hours and 100 mg/kg over 16 hours. NAC was diluted in 5% dextrose before administration. Patients in the control group received an identical volume of 5% dextrose placebo at the same infusion rate and duration.

Standard supportive management

All patients, irrespective of group allocation, received standardized supportive care in accordance with institutional toxicology protocols. This included early gastric lavage with potassium permanganate (1:10,000) when presentation occurred within one hour of ingestion, aggressive intravenous fluid resuscitation with balanced crystalloids, and supplemental oxygen therapy. Hemodynamic instability was defined as a mean arterial pressure < 65 mmHg despite adequate fluid resuscitation. Norepinephrine was administered as the first-line vasoactive agent in patients meeting this criterion.

Outcome measures and monitoring

In-hospital mortality was the primary outcome measure. Secondary outcomes included the requirement for inotropic support at admission and at 24 hours, as well as physiological and biochemical parameters measured at 24 hours, including systolic blood pressure, arterial pH, serum ALT, AST, and CK-MB. All secondary outcomes were prespecified in the study protocol. No adjustment for multiple comparisons was planned; therefore, analyses of secondary outcomes were considered exploratory. Adverse drug reactions were actively monitored throughout the infusion period. Prespecified adverse events of special interest included anaphylactoid reactions, such as flushing, urticaria, bronchospasm, and transient hypotension, as well as nausea, vomiting, and infusion-site reactions.

Sample size calculation

A priori sample size estimation was performed using the two-proportion z-test. Based on institutional data from confirmed zinc phosphide poisoning cases over the preceding three years, baseline in-hospital mortality was estimated at approximately 40%. Assuming a reduction in mortality to 15% with NAC, with a two-sided alpha of 0.05 and 80% power, the required sample size was approximately 47 participants per group. As only 30 participants per group were enrolled, the study should be interpreted as exploratory and underpowered for the primary endpoint.

Statistical analysis

Data were analyzed using SPSS Statistics software, Version 28.0 (IBM Corp., Armonk, NY). The normality of continuous variables was assessed using the Shapiro-Wilk test. Normally distributed continuous variables are presented as mean ± standard deviation (SD) and were analyzed using the Student’s t-test. Non-normally distributed variables are presented as median with interquartile range (IQR) and were analyzed using the Mann-Whitney U test. Categorical variables are presented as frequencies and percentages and were analyzed using the chi-square test or Fisher’s exact test, as appropriate. Relative risk (RR) with 95% confidence intervals (CI) was calculated for the primary outcome. There were no missing outcome data. No adjustment for multiple comparisons was performed, and secondary analyses were considered exploratory. Given the randomized design and comparable baseline characteristics, no post-hoc covariate adjustment was conducted. A p-value < 0.05 was considered statistically significant.

## Results

Participant characteristics

Sixty patients meeting the eligibility criteria were enrolled and allocated equally into two groups (30 patients in the NAC intervention arm and 30 in the placebo control arm). There was no loss to follow-up, and none of the participants dropped out. The overall study population had a mean age of 33 ± 15 years, with a male predominance (63%). Baseline demographic and clinical characteristics were comparable between the two groups. The exact quantity of toxin ingested could not be reliably quantified, which is a recognized limitation in toxicology studies. Baseline severity was assessed using initial hemodynamic status, requirement for inotropic support at admission, arterial pH, liver enzyme levels, and CK-MB levels. Table 1 presents the baseline demographic characteristics and sex distribution of the study population.

**Table 1:** Baseline demographic characteristics of the study population SD: standard deviation

Parameter	NAC group (n = 30)	Placebo group (n = 30)	Total (n = 60)
Mean age, years, ± SD	—	—	33 ± 15
Male, n	19	19	38 (63%)
Female, n	11	11	22 (37%)
Mode of poisoning	Suicidal (100%)	Suicidal (100%)	100%

Clinical presentation

The most common presenting complaint in both groups at admission was vomiting. Retrosternal chest pain, diffuse abdominal pain, shortness of breath, and altered sensorium were also frequently reported. These symptoms were similarly distributed between the study arms, with no statistically significant differences observed. In addition, there was no significant difference in the time from ingestion to hospital presentation between the NAC and placebo groups. Table 2 presents the distribution of presenting symptoms and time to hospital arrival in both study groups.

**Table 2:** Clinical presentation and time to hospital arrival ^*^P < 0.05 indicates statistical significance; ᵃPearson chi-square test; ᵇFisher’s exact test (used when expected cell count < 5). The test statistic is reported as χ² for the chi-square test; not applicable for Fisher’s exact test. All tests are two-tailed NAC: N-acetylcysteine

Variable	NAC (n = 30), n (%)	Placebo (n = 30), n (%)	Test statistic	P-value^*^
Vomitingᵃ	24 (80.0%)	22 (73.3%)	χ² = 0.30	0.58
Shortness of breathᵃ	12 (40.0%)	11 (36.7%)	χ² = 0.05	0.83
Altered sensoriumᵇ	6 (20.0%)	5 (16.7%)	—	0.75
Retrosternal painᵃ	21 (70.0%)	18 (60.0%)	χ² = 0.91	0.34
Abdominal painᵃ	13 (43.3%)	10 (33.3%)	χ² = 0.63	0.42
Time < 3 hrsᵃ	7 (23.3%)	8 (26.7%)	χ² = 0.07	0.79

Hemodynamic outcomes

The proportion of patients requiring inotropic support at admission was similar between the groups (30.0% vs. 26.7%; p = 0.83) (Table 3). At 24 hours, fewer patients in the NAC group continued to require inotropic support compared with the placebo group (6.7% vs. 23.3%; Fisher’s exact p = 0.145; RR: 0.29, 95% CI: 0.07-1.27; absolute risk reduction (ARR): 16.6%), although this difference did not reach statistical significance. Hemodynamic stabilization was defined as the resolution of the need for vasoactive support.

**Table 3:** Requirement for inotropic support ^*^P < 0.05 indicates statistical significance; ᵃPearson chi-square test; ᵇFisher’s exact test (used when expected cell count < 5). The test statistic is reported as χ² for the chi-square test; not applicable for Fisher’s exact test. All tests are two-tailed NAC: N-acetylcysteine

Variable	NAC (n = 30), n (%)	Placebo (n = 30), n (%)	Test statistic	P-value^*^
At admission	9 (30.0%)	8 (26.7%)	χ² = 0.04	0.83
At 24 hours	2 (6.7%)	7 (23.3%)	—	0.145

Biochemical parameters

Serum ALT and AST were significantly lower in patients receiving NAC compared to placebo (p < 0.001 for both). No significant difference was observed in arterial pH or CK-MB levels (p = 0.164 for CK-MB) (Table 4).

**Table 4:** Comparison of clinical and biochemical parameters between NAC and placebo groups ^*^P < 0.05 indicates statistical significance Student’s t-test was used for normally distributed variables (SBP, pH, CK-MB). Mann–Whitney U test was used for non-normally distributed variables (ALT, AST). Fisher’s exact test was used for the categorical variable (mortality) due to the small sample size. All tests are two-tailed NAC: N-acetylcysteine; SBP: systolic blood pressure; SD: standard deviation; ALT: alanine aminotransferase; IQR: interquartile range; AST: aspartate aminotransferase; CK-MB: creatine kinase-MB; RR: relative risk; CI: confidence interval

Parameter	NAC (n = 30)	Placebo (n = 30)	Test statistic	P-value^*^
SBP, mmHg, mean ± SD	105 ± 12	108 ± 11	t = -1.02	0.31
Arterial pH, mean ± SD	7.38 ± 0.08	7.35 ± 0.10	t = 1.29	0.20
ALT, IU/L, median (IQR)	92 (80-110)	205 (180-250)	U = 210	< 0.001
AST, IU/L, median (IQR)	48 (40-60)	150 (120-190)	U = 198	< 0.001
CK-MB, IU/L, mean ± SD	39 ± 6	42 ± 8	t = -1.41	0.164
Mortality, n (%)	2 (6.7%)	6 (20.0%)	Fisher's exact	0.254; RR: 0.33 (95% CI: 0.07-1.52)

Primary outcome: mortality

In-hospital mortality was lower in the NAC group than in the placebo group (two deaths (6.7%) vs. six deaths (20.0%); Fisher's exact p = 0.254). The absolute risk reduction was 13.3%; the relative risk was 0.33 (95% CI 0.07-1.52). This difference did not reach statistical significance at α = 0.05. Comparative analysis between the NAC and placebo groups showed lower mortality in the NAC group (6.7% vs. 20.0%); however, the difference did not reach statistical significance (Fisher's exact p = 0.254). Liver enzymes (ALT and AST) were significantly lower in the NAC group (p < 0.001), while no significant differences were observed in arterial pH or CK-MB levels (Table 4).

Prognostic comparison between survivors and non-survivors

Clinical and biochemical parameters were compared between survivors and non-survivors to identify factors associated with mortality. Non-survivors showed significantly greater hemodynamic instability, metabolic acidosis, hepatic injury, and myocardial injury than survivors. These differences were statistically significant across all measured parameters. Table 5 presents the comparison of systolic blood pressure, arterial pH, ALT, AST, and CK-MB levels between survivors and non-survivors.

**Table 5:** Comparison of parameters between survivors and non-survivors *P < 0.05 indicates statistical significance Student’s t-test was used for normally distributed variables (SBP, pH, CK-MB). Mann–Whitney U test was used for non-normally distributed variables (ALT, AST). All tests are two-tailed SBP: systolic blood pressure; SD: standard deviation; ALT: alanine aminotransferase; IQR: interquartile range; AST: aspartate aminotransferase; CK-MB: creatine kinase-MB

Parameter	Survivors (n = 52)	Non-survivors (n = 8)	Test statistic	P-value^*^
SBP, mmHg, mean ± SD	108 ± 10	72 ± 7	t = 9.1	<0.001
ALT, IU/L, median (IQR)	95 (80–120)	260 (180–320)	U = 45	<0.001
AST, IU/L, median (IQR)	60 (45–80)	240 (180–290)	U = 40	<0.001
pH, mean ± SD	7.38 ± 0.08	7.10 ± 0.15	t = 5.8	<0.001
CK-MB, IU/L, mean ± SD	40 ± 6	72 ± 9	t = 8.2	<0.001

Safety and adverse events

Active monitoring throughout the intervention period identified no adverse drug reactions attributable to NAC. No anaphylactoid reactions (urticaria, flushing, bronchospasm, or hypotension), nausea, vomiting, or infusion-site reactions were recorded. No patient required discontinuation of the study infusion.

## Discussion

This preliminary single-center trial demonstrated that intravenous NAC as an adjunct to standard supportive care was associated with a clinically meaningful but statistically non-significant trend toward lower in-hospital mortality and reduced inotropic support requirements in acute zinc phosphide poisoning. These findings are hypothesis-generating and are not sufficient to change clinical practice; larger multicenter randomized trials are required to determine whether the observed trend reflects a true treatment effect. Zinc phosphide poisoning remains a major toxicological emergency with high fatality and no specific antidote, with management relying on early recognition, resuscitation, and intensive supportive care [1,19].

Previous reviews of aluminum phosphide poisoning similarly emphasize that treatment remains largely supportive, as no definitive antidote has been established [20,21]. Phosphine inhibits mitochondrial oxidative phosphorylation and induces severe oxidative stress, leading to cellular energy failure and multiorgan dysfunction [6,9]. Oxidative stress and mitochondrial injury are key therapeutic targets in this context [10]. NAC, through its antioxidant and cytoprotective properties, acts as a glutathione precursor and free-radical scavenger [7].

The current findings are directionally consistent with experimental data and prior clinical studies of NAC in phosphide poisoning. Experimental models have demonstrated that NAC can reduce lipid peroxidation and preserve mitochondrial integrity [7,9]. Clinical studies in aluminum phosphide poisoning have reported improved survival and reduced vasopressor requirements with NAC [8,14]. The observed reduction in inotropic support at 24 hours in the NAC group, although not statistically significant, may still be clinically relevant, as refractory shock is a leading cause of death in severe phosphide poisoning [5].

Reported clinical complications of phosphide poisoning include severe circulatory failure and hematological abnormalities, while successful case management has generally relied on prompt intensive supportive care [22,23]. However, these findings should be interpreted cautiously, as the study was underpowered for clinical outcomes. The significant reductions in ALT and AST in the NAC group are consistent with the hepatoprotective properties of NAC, including glutathione replenishment, oxidative stress reduction, and inflammatory modulation [10]. These findings align with prior reports of reduced hepatic injury with NAC in phosphide and other poisonings [17,18]. Because these biochemical outcomes were secondary endpoints, they should be interpreted as supportive rather than definitive evidence of clinical benefit.

While zinc phosphide and aluminum phosphide share a common phosphine-mediated mechanism of toxicity, zinc ions released from zinc phosphide may contribute additional direct cytotoxic effects, potentially modifying the response to antioxidant therapy. Not all prior studies of NAC in phosphide poisoning have demonstrated a mortality benefit, particularly smaller observational studies subject to confounding. The present results are directionally consistent with randomized data from aluminum phosphide poisoning [13,14,18], but extrapolation across phosphide compounds remains uncertain; therefore, these findings should be considered specific to zinc phosphide until confirmed in larger studies.

No significant differences were observed in arterial pH or CK-MB levels. The lack of significant change in arterial pH may reflect pre-hospital factors, including toxin dose and delay in presentation, which substantially influence acid-base status before treatment. Differences in CK-MB levels may have been attenuated by the multifactorial nature of myocardial injury in phosphide poisoning, which involves metabolic derangements, electrolyte disturbances, and direct cellular toxicity beyond ischemic mechanisms [16]. The safety profile of NAC was favorable, with no adverse drug reactions observed. This is consistent with the established literature indicating that NAC is generally well tolerated even at high doses in toxicological emergencies [7]. However, the sample size was small, and the study was not powered to detect rare adverse events.

Limitations and future research directions

This study has several limitations. It was conducted at a single tertiary care center with a relatively small sample size (n = 60), which limits external validity and the ability to detect rare adverse events or perform subgroup analyses. The enrolled sample size was smaller than that estimated in the a priori sample size calculation, and consequently, the study may have been underpowered to detect a statistically significant difference in the primary outcome. Only short-term in-hospital outcomes were evaluated; long-term hepatic, cardiac, or neurological sequelae were not assessed. The quantity of toxin ingested could not be reliably measured, introducing variability in poisoning severity despite randomization. Pre-hospital delays and variability in initial management represent potential confounders that were not fully controlled. The study was not stratified by poisoning severity at presentation, and mechanistic biomarkers of oxidative stress and mitochondrial dysfunction were not measured, limiting the ability to directly attribute observed clinical trends to NAC’s antioxidant mechanism. No adjustment for multiple comparisons was performed; therefore, secondary outcomes should be interpreted as exploratory.

Future research should focus on multicenter, large randomized trials with stratification by poisoning severity and time of presentation. Long-term follow-up is needed to evaluate persistent hepatic, cardiac, neurological, and functional outcomes. Standardized supportive care protocols across centers would reduce variability. Future studies should incorporate mechanistic biomarkers of oxidative stress and mitochondrial dysfunction to validate NAC’s therapeutic mechanism. Research on optimal NAC dosing and combination antioxidant therapy is also warranted.

## Conclusions

In this preliminary single-center trial, adjunctive intravenous NAC showed a clinically meaningful but statistically non-significant trend toward lower in-hospital mortality and reduced inotropic support requirements in acute zinc phosphide poisoning. These findings are hypothesis-generating and are not sufficient to change clinical practice; larger multicenter randomized trials are required to determine whether NAC provides a statistically significant and clinically meaningful benefit. NAC’s antioxidant and cytoprotective properties represent a plausible mechanism for attenuating phosphine-mediated cellular damage. The observed significant reduction in hepatic enzyme levels provides supportive biochemical evidence. Its favorable safety profile and wide availability support further investigation. Larger multicenter studies should optimize dosing schedules and evaluate NAC across a range of poisoning severities.
